# Supplementary material for: Mechanical Improvement of Graphene Oxide Film via the Synergy of Intercalating Highly Oxidized Graphene Oxide and Borate Bridging
Source: Nanomaterials (Basel). 2025 Apr 20;15(8):630. doi: 10.3390/nano15080630 (PMC12029253; doi:10.3390/nano15080630)
Supplement: Supplementary file 1 [file nanomaterials-15-00630-s001.zip › nanomaterials-3572343-supplementary.pdf]

Supplementary Information

Mechanical improvement of graphene oxide film via the synergy of intercalating high oxidized graphene oxide and borate bridging

Yiwei Quan [1,2], Peng He [1,2,*], Guqiao Ding [1,2,*]

[1] State Key Laboratory of Functional Materials for Informatics, Shanghai Institute of Microsystem and Information Technology, Chinese Academy of Sciences, 865 Changning Road, Shanghai 200050, P. R. China

[2] College of Materials Science and Opto-Electronic Technology, University of Chinese Academy of Sciences, Beijing, 100049, PR China

*Corresponding authors. E-mails: hepeng@mail.sim.ac.cn, gqding@mail.sim.ac.cn.

Supplementary Figure

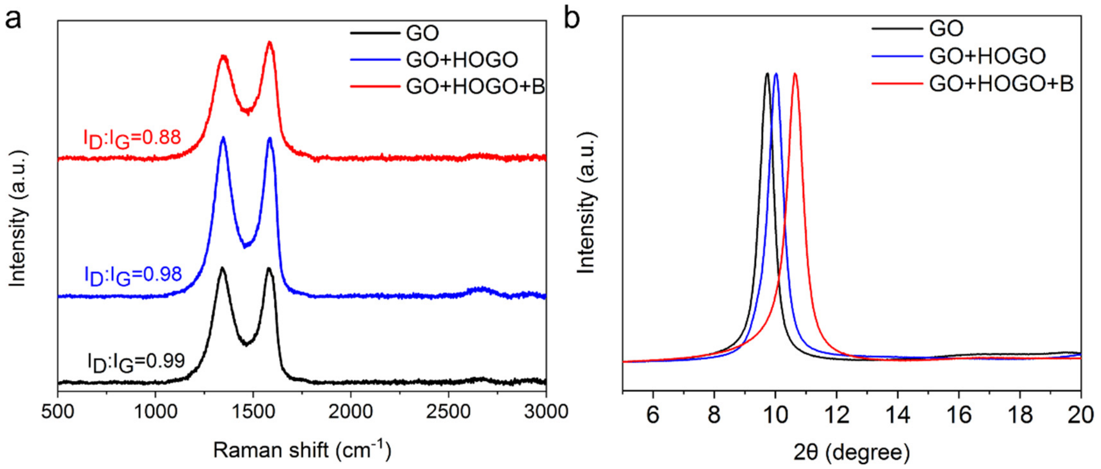

Figure S1. Characterization of the GO-based films. (a) Raman spectra of GO, GO+HOGO, GO+HOGO+B films. The D/G ratio of GO, GO+HOGO, GO+HOGO+B films are 0.99, 0.98 and 0.88, respectively. (b) X-ray diffraction (XRD) of GO, GO+HOGO, GO+HOGO+B films.

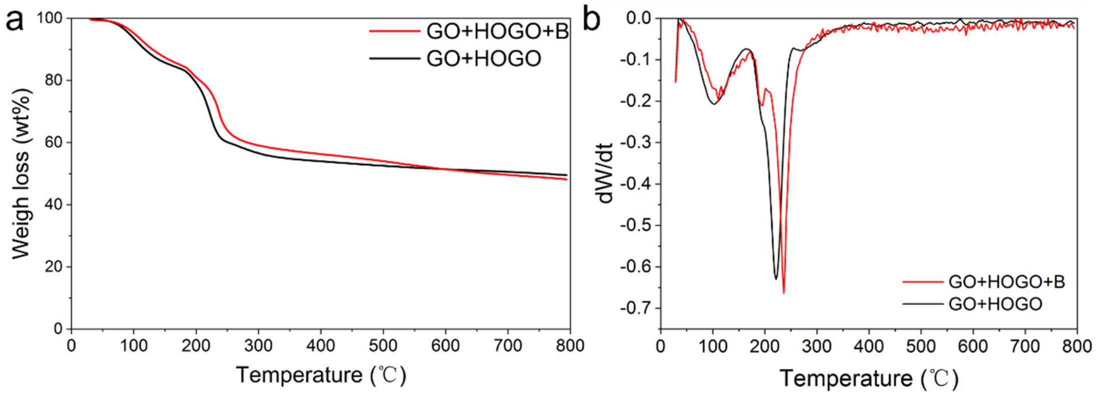

Figure S2. Thermogravimetric (TG) analysis (a) TG curves of borate-crosslinking GO+HOGO films (red) and GO+HOGO films (black), respectively. (b) DTG curves of borate-crosslinking GO+HOGO films (red) and GO+HOGO films (black), respectively.

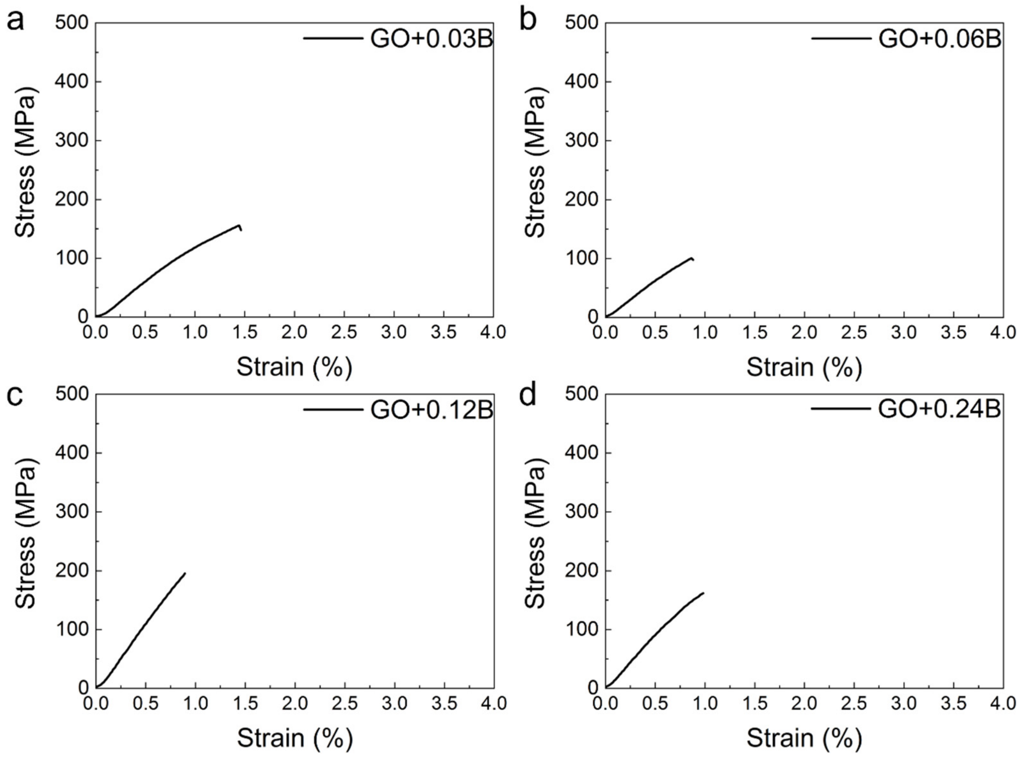

Figure S3. Tensile stress-strain curves for different GO films. (a-d) GO+B films.

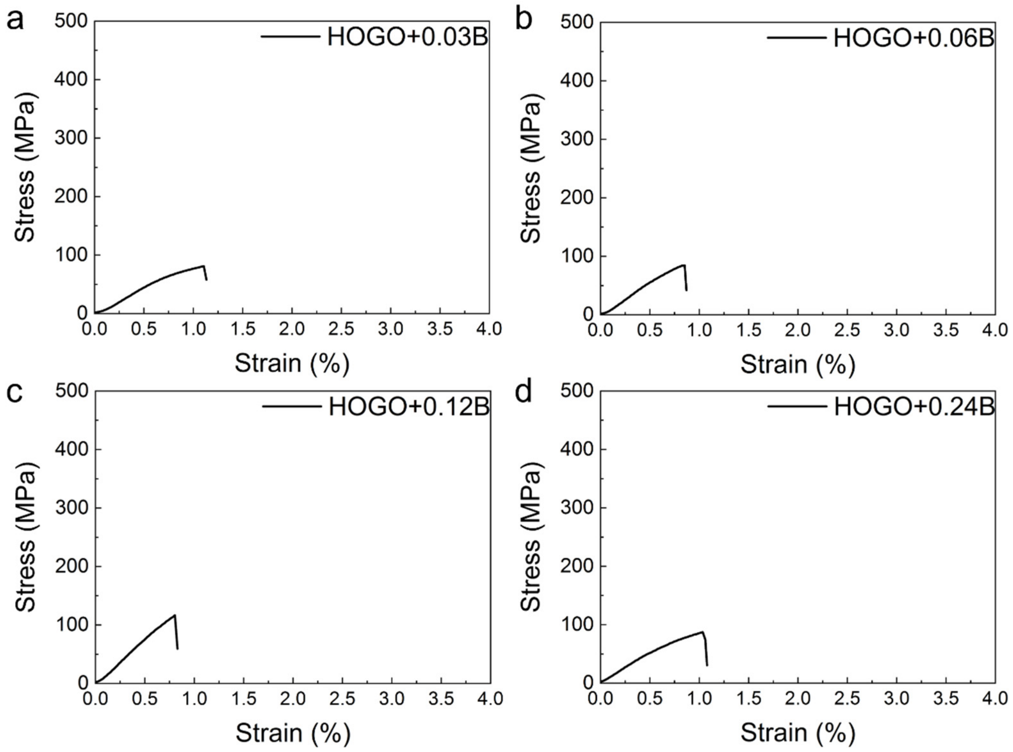

Figure S4. Tensile stress-strain curves for different GO films. (a-d) HOGO+B films.

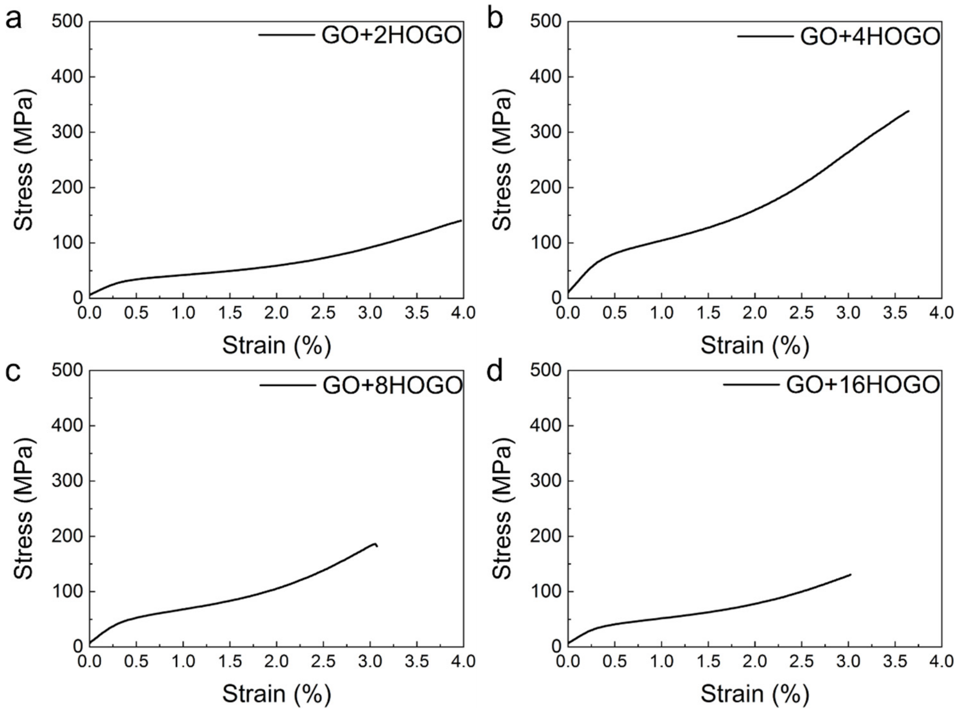

Figure S5. Tensile stress-strain curves for different GO films. (a-d) GO+HOGO films.

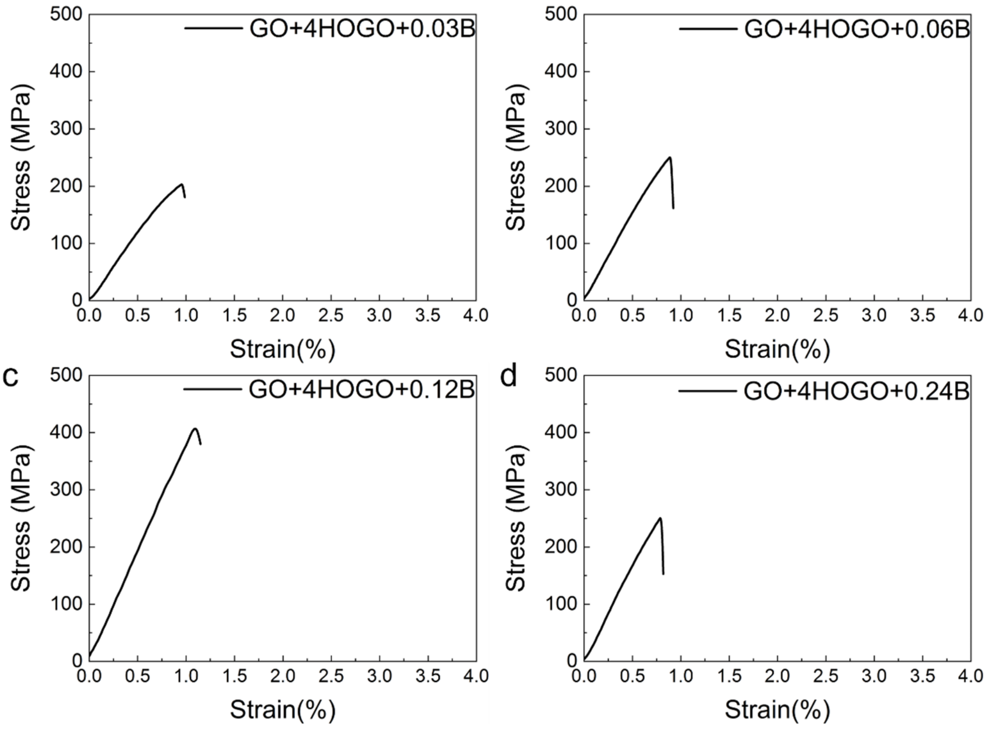

Figure S6. Tensile stress-strain curves for different GO films. (a-d) GO+HOGO+B films.

Table S1. Mechanical properties of different GO films.

Sample	Modulus (GPa)	Tensile strength (MPa)	Strain (%)	Toughness (MJ m^{-3})
GO	13.5	216.5	2.330	2.6
HOGO	7.9	46.6	1.750	0.6
GO+2HOGO	8.8	140.3	3.970	2.7
GO+4HOGO	22.0	338.3	3.643	6.0
GO+8HOGO	15.2	182.2	3.071	2.9
GO+16HOGO	11.2	130.8	3.023	2.1
GO+0.03B	15.2	155.7	1.445	1.2
GO+0.06B	14.1	100.4	0.864	0.5
GO+0.12B	27.4	191.0	0.876	0.9
GO+0.24B	22.3	161.8	0.981	0.9
HOGO+0.03B	11.3	81.0	1.106	0.5
HOGO+0.06B	14.2	84.5	0.852	0.4
HOGO+0.12B	18.7	116.7	0.803	0.5
HOGO+0.24B	12.1	87.5	1.034	0.6
GO+4HOGO+0.03B	29.1	205.0	0.964	1.1
GO+4HOGO+0.06B	33.4	253.6	0.899	1.3
GO+4HOGO+0.12B	43.8	417.2	1.103	2.5
GO+4HOGO+0.24B	37.6	254.2	0.802	1.1

Table S2. Mechanical properties of GO+HOGO, borate-crosslinking GO+HOGO films and other natural materials, GO-based materials, rGO-based materials in modulus, tensile strength and toughness, including GO and rGO films linked by ionic bonding, covalent bonding and synergistic enhancement.

	**Reference**	**Materials**	**Modulus (GPa)**	**Tensile strength (MPa)**	**Strain (%)**	**Toughness (MJ m^{-3})**
Natural Materials	[1]	Nacre	26.0-42.0	200.0	0.5	2.6
	[1]	Bone	10.0-15.0	50.0-200.0	-	2.0-10.0
	[2]	Bamboo	20.0-40.0	-	-	-
GO-based Materials	[3]	GO+Ca^{2+}	28.1 ± 1.2	125.8	0.4	0.3
		GO+Mg^{2+}	27.9 ± 1.8	87.9	0.5	0.1
	[4]	GO+Zn^{2+}	35.2	142.2	0.4	0.3
	[5]	GO+PAA	33.3 ± 2.7	91.9	0.3	0.2
	[6]	GO+GA	26.0.-34.7	101.0	0.4	0.3
	[7]	GO+PGO	35.1 ± 1.7	119.0 ± 27.0	0.4 ± 0.1	0.4
	[8]	GO+B	127.0 ± 4.0	185.0	0.1	0.1
	[9]	GO+B	109.9	135.0	0.1	0.1
	[10]	GO+PDA+PEI	84.8 ± 2.9	179.0	0.2	0.2
	[11]	GO+PVA	25.3 ± 0.5	255.7	2.1	2.5
	[12]	GO+PDA	11.3 ± 4.5	175.0	1.5	1.5
	[13]	GO+PVA	40.3	80.2	0.3	0.1
		GO+PMMA	10.1	148.3	3.2	2.4
	[14]	GO+Al^{3+}	26.2 ± 4.6	120.0	0.5	0.2
	Our work	LOGO+HOGO	22.0	338.3	3.6	6.0
		LOGO+HOGO+B	43.8	417.2	1.1	2.5
rGO-based Materials	[15]	rGO-PVA	10.4	188.9	2.7	2.5
	[16]	rGO–PCDO	1.6	129.6	8.0	3.9
	[17]	rGO–PAPB	8.8	382.0	4.3	7.5
	[12]	rGO–PDA	4.1	204.9	5.0	4.0
	[18]	rGO-CS	5.3	526.7	10.0	17.7
	[19]	rGO-CMC-Mn^{2+}	12.6	475.2	3.8	6.6
	[20]	rGO-PSE-AP-Cr^{3+}	11.7	821.0	7.0	20.0
	[21]	rGO-BPDD	16.2	1054.0	6.5	36.0
	[22]	rGO-BP-AD	3.8	653.5	17	51.8
	[23]	rGO-stretch	62.8	1100.0	1.6	-
	[24]	rGO-PSE-AP-PCO-stretch	64.5	1,547.0	3.7	35.9

References

1. Wegst, U.G.; Bai, H.; Saiz, E.; Tomsia, A.P.; Ritchie, R.O. Bioinspired Structural Materials. *Nat. Mater.* **2015**, *14*, 23-36, doi:10.1038/nmat4089.
2. Wegst, U.G.K. Bamboo and Wood in Musical Instruments. *Annu. Rev. Mater. Res.* **2008**, *38*, 323-349, doi:10.1146/annurev.matsci.38.060407.132459.
3. Park, S.; Lee, K.S.; Bozoklu, G.; Cai, W.; Nguyen, S.T.; Ruoff, R.S. Graphene oxide papers modified by divalent ions-enhancing mechanical properties via chemical cross-linking. *ACS Nano* **2008**, *2*, 572-578, doi:10.1021/nn700349a.
4. Lam do, V.; Gong, T.; Won, S.; Kim, J.H.; Lee, H.J.; Lee, C.; Lee, S.M. A robust and conductive metal-impregnated graphene oxide membrane selectively separating organic vapors. *Chem. Commun.* **2015**, *51*, 2671-2674, doi:10.1039/c4cc08896d.
5. Park, S.; Dikin, D.A.; Nguyen, S.T.; Ruoff, R.S. Graphene Oxide Sheets Chemically Cross-Linked by Polyallylamine. *J. Phys. Chem. C.* **2009**, *113*, 15801-15804, doi:10.1021/jp907613s.
6. Gao, Y.; Liu, L.Q.; Zu, S.Z.; Peng, K.; Zhou, D.; Han, B.H.; Zhang, Z. The effect of interlayer adhesion on the mechanical behaviors of macroscopic graphene oxide papers. *ACS Nano* **2011**, *5*, 2134-2141, doi:10.1021/nn103331x.
7. Mao, L.; Park, H.; Soler-Crespo, R.A.; Espinosa, H.D.; Han, T.H.; Nguyen, S.T.; Huang, J. Stiffening of graphene oxide films by soft porous sheets. *Nat. Commun.* **2019**, *10*, 3677-3685, doi:10.1038/s41467-019-11609-8.
8. An, Z.; Compton, O.C.; Putz, K.W.; Brinson, L.C.; Nguyen, S.T. Bio-inspired borate cross-linking in ultra-stiff graphene oxide thin films. *Adv. Mater.* **2011**, *23*, 3842-3846, doi:10.1002/adma.201101544.
9. Liu, S.Y.; Hu, K.W.; Cerruti, M.; Barthelat, F. Ultra-stiff graphene oxide paper prepared by directed-flow vacuum filtration. *Carbon* **2020**, *158*, 426-434, doi:10.1016/j.carbon.2019.11.007.
10. Tian, Y.; Cao, Y.; Wang, Y.; Yang, W.; Feng, J. Realizing ultrahigh modulus and high strength of macroscopic graphene oxide papers through crosslinking of mussel-inspired polymers. *Adv. Mater.* **2013**, *25*, 2980-2983, doi:10.1002/adma.201300118.
11. Liu, L.; Gao, Y.; Liu, Q.; Kuang, J.; Zhou, D.; Ju, S.; Han, B.; Zhang, Z. High mechanical performance of layered graphene oxide/poly(vinyl alcohol) nanocomposite films. *Small* **2013**, *9*, 2466-2472, doi:10.1002/smll.201300819.
12. Cui, W.; Li, M.; Liu, J.; Wang, B.; Zhang, C.; Jiang, L.; Cheng, Q. A strong integrated strength and toughness artificial nacre based on dopamine cross-linked graphene oxide. *ACS Nano* **2014**, *8*, 9511-9517, doi:10.1021/nn503755c.
13. Putz, K.W.; Compton, O.C.; Palmeri, M.J.; Nguyen, S.T.; Brinson, L.C. High-Nanofiller-Content Graphene Oxide-Polymer Nanocomposites via Vacuum-Assisted Self-Assembly. *Adv. Funct. Mater.* **2010**, *20*, 3322-3329, doi:10.1002/adfm.201000723.
14. Yeh, C.N.; Raidongia, K.; Shao, J.; Yang, Q.H.; Huang, J. On the origin of the stability of graphene oxide membranes in water. *Nat. Chem.* **2014**, *7*, 166-170, doi:10.1038/nchem.2145.
15. Li, Y.Q.; Yu, T.; Yang, T.Y.; Zheng, L.X.; Liao, K. Bio-inspired nacre-like composite films based on graphene with superior mechanical, electrical, and biocompatible properties.

Adv. Mater. Int. **2012**, *24*, 3426-3431, doi:10.1002/adma.201200452.

16. Cheng, Q.; Wu, M.; Li, M.; Jiang, L.; Tang, Z. Ultratough artificial nacre based on conjugated cross-linked graphene oxide. *Angew. Chem. Int. Ed. Engl.* **2013**, *52*, 3750-3755, doi:10.1002/anie.201210166.
17. Zhang, M.; Huang, L.; Chen, J.; Li, C.; Shi, G. Ultratough, ultrastrong, and highly conductive graphene films with arbitrary sizes. *Adv. Mater.* **2014**, *26*, 7588-7592, doi:10.1002/adma.201403322.
18. Wan, S.; Peng, J.; Li, Y.; Hu, H.; Jiang, L.; Cheng, Q. Use of Synergistic Interactions to Fabricate Strong, Tough, and Conductive Artificial Nacre Based on Graphene Oxide and Chitosan. *ACS Nano* **2015**, *9*, 9830-9836, doi:10.1021/acsnano.5b02902.
19. Gong, S.; Zhang, Q.; Wang, R.; Jiang, L.; Cheng, Q. Synergistically toughening nacre-like graphene nanocomposites via gel-film transformation. *J. Mater. Chem. A* **2017**, *5*, 16386-16392, doi:10.1039/c7ta03535g.
20. Wan, S.; Fang, S.; Jiang, L.; Cheng, Q.; Baughman, R.H. Strong, Conductive, Foldable Graphene Sheets by Sequential Ionic and pi Bridging. *Adv. Mater. Int.* **2018**, e1802733, doi:10.1002/adma.201802733.
21. Wan, S.J.; Chen, Y.; Wang, Y.L.; Li, G.W.; Wang, G.R.; Liu, L.Q.; Zhang, J.Q.; Liu, Y.Z.; Xu, Z.P.; Tomsia, A.P.; et al. Ultrastrong Graphene Films via Long-Chain π-Bridging. *Matter* **2019**, *1*, 389-401, doi:10.1016/j.matt.2019.04.006.
22. Zhou, T.; Ni, H.; Wang, Y.; Wu, C.; Zhang, H.; Zhang, J.; Tomsia, A.P.; Jiang, L.; Cheng, Q. Ultratough graphene-black phosphorus films. *Proc. Natl. Acad. Sci. U.S.A.* **2020**, *117*, 8727-8735, doi:10.1073/pnas.1916610117.
23. Li, P.; Yang, M.; Liu, Y.; Qin, H.; Liu, J.; Xu, Z.; Liu, Y.; Meng, F.; Lin, J.; Wang, F.; et al. Continuous crystalline graphene papers with gigapascal strength by intercalation modulated plasticization. *Nat. Commun.* **2020**, *11*, 2645, doi:10.1038/s41467-020-16494-0.
24. Wan, S.; Chen, Y.; Fang, S.; Wang, S.; Xu, Z.; Jiang, L.; Baughman, R.H.; Cheng, Q. High-strength scalable graphene sheets by freezing stretch-induced alignment. *Nat. Mater.* **2021**, *20*, 624-631, doi:10.1038/s41563-020-00892-2.
